# Pharmacoeconomic Analysis of Heartworm Preventive Compliance and Revenue in Veterinary Practices in the United States

**DOI:** 10.3389/fvets.2021.602622

**Published:** 2021-05-21

**Authors:** Kennedy Mwacalimba, Deborah Amodie, Lisa Swisher, Marina Moldavchuk, Christopher Brennan, Claire Walther, Kelly Bowman

**Affiliations:** ^1^Zoetis, Parsippany, NJ, United States; ^2^Covetrus, Portland, ME, United States

**Keywords:** heartworm (*Dirofilaria immitis*), dog, injectable moxidectin, prevention, compliance

## Abstract

**Background:** Heartworm disease (HWD) is a potentially fatal condition caused by the nematode *Dirofilaria immitis*. It is endemic in North America, and the American Heartworm Society recommends that owned dogs be on a Food and Drug Administration-approved HWD preventive year-round. The objective of this study was to compare the 12-month HWD preventive purchase compliance rates of injectable moxidectin (ProHeart® 6) and the dose equivalent in monthly HWD preventives and their associated economic value to the veterinary hospital.

**Methods:** This study used retrospective anonymized transactional data of 7,926,392 unique dogs from 3,737 companion animal practices across the US for the period 2014–2017. Compliance was defined using American Heartworm Society guidelines. Comparisons were purchases of a 6-month moxidectin injection or six doses of any monthly HWD or HWD combination preventive product, tracked for the next preventive purchase 5–7 months later. Total revenue, HWD prevention cost, 12-month repurchase compliance, and patient retention were calculated. Data were expressed on an annualized basis. Compliance comparisons were calculated based on proportion analysis with the SAS ProbNorm function (SAS 9.4, Cary, NC), using a two-sided *t*-test, at the 5% level of significance (*P* < 0.05).

**Results:** At 51.7%, annual compliance with injectable moxidectin was higher than the dose equivalent in monthly HWD preventives, which was 24.4% (*P* = 0.0001). Eighty-five percent of patients on injectable moxidectin recorded additional transactions during the first visit (average invoice of $161), compared with only 55% of pet owners who purchased monthly HWD prevention (average invoice $141) or monthly HWD combination (average invoice of $171). The average costs of 6 months of HWD preventives were as follows: injectable moxidectin, $48 (29.7% of the total visit invoice); monthly HWD prevention, $45 (31.0% of the total invoice); and monthly HWD combination, 95 (55.6% of the total visit invoice). Finally, dogs receiving injectable moxidectin had a higher proportion of patients with repeat injections within 12 months between 2014 and 2017, with 68% retention rate after 4 years. In comparison, the six-dose monthly HWD cohort retention rate dropped to 55% by 2017.

**Conclusions:** Dogs receiving injectable moxidectin had higher HWD preventive compliance, generated more practice revenue, and had a higher rate of practice retention compared with monthly HWD products.

## Introduction

Heartworm disease (HWD) is a potentially fatal condition caused by the nematode *Dirofilaria immitis* and is perhaps the most important parasite of dogs in North America (1). Heartworm-positive dogs have been reported in every state on the continental US (2, 3). The 2018 American Heartworm Society (AHS) guidelines recommend that all owned dogs be on a Food and Drug Administration (FDA)-approved HWD preventive year-round (4). Prevention of HWD in dogs relies on a single drug class, the macrocyclic lactones (1). In the US, there are three common forms of HWD prevention: monthly oral tablets/chews, monthly topical liquids, and an injectable suspension (ProHeart® hereafter referred to as “injectable moxidectin”).

The consistent use of effective HWD preventives is the primary goal in reducing the prevalence of heartworm disease infection in domestic dogs (5). This requires owner compliance (or adherence), which generally describes taking prescribed medication according to schedule (6). However, owner lack of compliance in purchasing and administering preventives occurs frequently and is believed to be the most common explanation for dogs developing adult heartworm infections (7). Furthermore, the recent observation of resistance to macrocyclic lactones in specific canine *D. immitis* strains in the US is a rising concern (8).

Achieving high owner compliance with preventive medications has been a challenge for veterinarians (9). There are, however, several barriers to pet owner compliance with HWD preventive administration. One study that examined knowledge of HWD among members of a national hunting dog club found that cost (33%) and administration (26%) were important barriers (10). Nevertheless, poor owner compliance has important health consequences. One review of US veterinary clinic medical records showed that in 81% of HWD cases, insufficient preventive doses were purchased, suggesting improving owner purchase compliance is key to mitigating the spread of this potentially deadly infectious disease of domestic dogs (11). Injectable moxidectin prevents infection by D. immitis for 6 or 12 months (ProHeart® 6 or ProHeart® 12, respectively) and is administered only by a veterinary professional, thus ensuring 100% compliance for the prescribed period of time. Because injectable moxidectin is administered by a veterinary professional, the veterinarian, and not the client, maintains control over the disease prevention process. Its use as a heartworm prophylactic therefore guarantees pet owner compliance with an efficacious HWD prevention protocol.

There is limited understanding of how HWD preventive selection impacts practice revenue and client retention over time. There are also very little data comparing purchase compliance between injectable, topical, and oral formulations of HWD preventive during a 12-month period (as per AHS guidelines).

In human medicine, retrospective databases are increasingly being used to describe the incidence and prevalence of medication compliance (12). The importance of compliance studies includes helping health providers understand how medication compliance varies among patients and how that variation could impact health outcomes (12). The purpose of this study was therefore to compare HWD preventive repurchase compliance, revenue, and retention between dogs receiving monthly preventive vs. biannual injectable moxidectin from their veterinarian each year. The economic value was summarized as the average revenue per dog per preventive visit each year, i.e., visit 1 and visit 2.

## Methods

This was a descriptive longitudinal pharmacoeconomic study. It followed the pharmacoeconomic guidelines and checklist for compliance and persistence studies using retrospective databases (12).

### Study Population

The study population was 7,926,392 unique dogs from 3,737, non-corporate, companion animal practices across the US.

### Selection Criteria

Criteria for inclusion in this study were as follows:

1. Dogs whose owners purchased at least six doses of a monthly HWD or HWD combination preventive product or received one 6-month moxidectin injection from their veterinarian each year for the time frame 2014–2017;
2. Dogs whose owners remained active clients in their respective veterinary practices over the entire observation period.

### Data Description

Data were collected from complete transaction records of products and services purchased by clients between January 2014 and December 2017. Medical records were not included in this study. Transaction records were sorted by purchase of a 6-month moxidectin injection or six doses of any monthly HWD or HWD combination preventive product. These patients were then tracked for their next HWD preventive purchase 5–7 months later. The 7-month return visit cutoff was selected because macrocyclic lactones have a 30-day reach back effect (13). Dogs were followed as injectable moxidectin, monthly HWD, or HWD combination preventive cohorts starting from January 1, 2014.

### Data Analysis

The AHS guidelines were used to define compliance for 12 months (4). Data were summarized using Microsoft Excel™. Compliance comparisons and results were based on proportion analysis with the SAS ProbNorm function (SAS 9.4, Cary, NC), using a two-sided test, at the 5% level of significance (*P* < 0.05).

#### Twelve-Month Compliance

The period of observation for compliance was the full 12 months of each calendar year. A dog was defined as compliant with extended release moxidectin dosing if it received a 6-month moxidectin injection followed by a second injection within 5–7 months of the first. Dogs were sorted according to the compliance categories stated below:

- Early (within 1–4 months of the first injection).
- On time, i.e., 12 months compliant (within 5–7 months of the first injection).
- Late (within 8–12 months of the first injection).
- Non-compliant (did not receive a second injection during the 12-month period).

A patient was considered *purchase*-compliant for monthly HWD or HWD combination preventives if it included an initial recorded purchase of six doses, followed by a second transaction for six doses within 5–7 months of the first. Like injectable moxidectin dogs, the period of observation for compliance was 12 months. Repurchases of six doses of a monthly HWD or HWD combination preventive could be made:

- Early (within 1–4 months of the first six-dose purchase).
- On time, i.e., 12 months compliant (within 5–7 months of the first six-dose purchase).
- Late (within 8–12 months of the first six-dose purchase).
- Non-compliant (did not repurchase their HWD preventive during the 12-month period).

#### Total Revenue per HWD Preventive Visit

Total revenue per visit was calculated for each transaction with a 6-month injectable moxidectin purchase or six doses of a monthly HWD or HWD combination product. Totals included revenues from all products and services purchased during the visit. The total amount was then averaged by the number of dogs in each cohort.

#### Cost of HWD Prevention

The average annual cost of HWD prevention per dog by preventive modality was determined for all dogs whose owners had purchased 6 months or more of HWD prevention in the calendar year.

#### Retention by Preventive Modality

Patient retention by HWD prevention modality was determined based on purchase of 12 or more doses each calendar year. The calculation was as follows: an injectable moxidectin recipient was counted as retained in the year if there was a follow-up injectable moxidectin transaction in the subsequent 12 months. Similarly, a monthly preventive recipient was considered retained in the year if there was a cohort product purchase in the subsequent 12 months. For monthly preventive dogs, the repurchase could be a different monthly preventive product from the first. For this comparison, dogs had to have records of 12 or more dose purchases of monthly HWD preventives or received two injections of moxidectin each year. All dogs were first followed as cohorts starting in 2014 and their transaction history followed through to 2017. We then observed the proportion of dogs retained over the duration of the study period.

The following patients were excluded:

- All initial HWD purchases for the year that were <6 doses.
- Patients that switched from moxidectin to monthly HWD or from monthly HWD to moxidectin between 2014 and 2017.

## Results

### Twelve-Month Compliance

Between 2014 and 2017, the proportion of dogs that were compliant with receiving two injections of moxidectin 5–7 months apart each year was 51.7% (range 49.7–53.1%). In comparison, the proportion of dogs that were compliant with two transactions for six doses of a monthly HWD product within 5–7 months was 24.4% for the same time period (range 24.2–24.6%). The difference in purchase compliance rates between injectable moxidectin and monthly HWD preventives was statistically significant for each year observed (Table 1).

**Table 1:** Twelve-month compliance comparison for injectable moxidectin vs. monthly HWD prevention.

**Year**	**All injectable moxidectin**	**Compliant**	**Percent**	**All six doses of monthly HWD**	**Compliant**	**Percent**	***P*-value**
2014	183,066	90,915	49.7%	1,719,665	415,384	24.2%	0.0001
2015	197,211	100,503	51.0%	1,743,727	424,161	24.3%	0.0001
2016	245,002	130,024	53.1%	1,714,466	417,301	24.3%	0.0001
2017	296,864	157,338	53.0%	1,685,452	414,970	24.6%	0.0001

Only 24.4% of monthly HWD or HWD combination preventive users (range 24.2–24.6%; Table 1) purchased exactly six doses within 5–7 months of the first six-dose purchase. When repurchases of over six doses or <6 doses were included, this proportion increased to 30.2% (range 29.9–30.3%; Table 2).

**Table 2:** Twelve-month purchase compliance for dogs receiving six doses of a monthly HWD or HWD combination product at first visit.

**Year**	***N***	**Compliant**	**Proportion**	**Compliant (>6 doses)**	**Proportion**	**Compliant (<6 doses)**	**Proportion**	**Combined proportion**
2014	1,719,665	415,384	24.2%	52,656	3.1%	45,370	2.6%	29.9%
2015	1,743,727	424,161	24.3%	56,645	3.2%	47,812	2.7%	30.2%
2016	1,714,466	417,301	24.3%	56,085	3.3%	47,641	2.8%	30.4%
2017	1,685,452	414,970	24.6%	47,893	2.8%	48,177	2.9%	30.3%

Of the moxidectin recipients, 1.4% received their second injection early, while 10% of dogs (range 8.9–10.5%) received their second injection late. In comparison, an average of 8.4% of dogs (range 8.3–8.6%) receiving monthly HWD or HWD combination product repurchased early, and 11.3% (range 11.0–11.6%) repurchased late (Table 3).

**Table 3:** Summary of the proportion of patients receiving doses early or late after initial purchase.

	**Year**	***N***	**Early (1–4 months of index visit)**	**Proportion**	**Late (8–12 months of index visit)**	**Proportion**
Biannual injectable moxidectin						
	2014	183,066	2,617	1.4%	16,233	8.9%
	2015	197,211	2,717	1.4%	20,323	10.3%
	2016	245,002	3,533	1.4%	25,600	10.4%
	2017	296,864	4,018	1.4%	31,063	10.5%
	Average	230,536	3,221	1.4%	23,305	10.0%
Six or more doses of monthly HWD or HWD combination preventive						
	2014	1,719,665	174,567	10.1%	238,966	13.9%
	2015	1,743,727	174,540	10.0%	239,494	13.8%
	2016	1,714,466	167,335	9.7%	230,772	13.4%
	2017	1,685,452	162,364	9.6%	218,876	13.0%
	Average	1,715,828	169,702	9.9%	232,027	13.5%
<6 doses of monthly HWD or HWD combination preventive						
	2014	1,719,665	29,842	1.7%	28,208	1.6%
	2015	1,743,727	29,952	1.7%	29,411	1.7%
	2016	1,714,466	28,839	1.7%	29,530	1.7%
	2017	1,685,452	28,921	1.7%	29,567	1.8%
	Average	1,715,828	29,389	1.7%	29,179	1.7%

Finally, 32% of moxidectin dogs received only one injection in a 12-month period (i.e., were non-compliant; range 31.0–33.1%) compared with 55.6% of dogs that had a single transaction for six doses of a monthly HWD product in a 12-month period (range 55.4–55.9%; Table 4).

**Table 4:** Compliance comparison: six doses of monthly HWD or HWD combination vs. biannual injectable moxidectin.

	**Year**	***N***	**Early**	**Compliant at 5–7 months**	**Late**	**Switched**	**12 months compliant**	**No repurchase in 12 months**
Biannual injectable moxidectin								
	2014	183,066	1.4%	49.7%	8.9%	6.9%	66.9%	33.1%
	2015	197,211	1.4%	51.0%	10.3%	6.3%	69.0%	31.0%
	2016	245,002	1.4%	53.1%	10.4%	3.1%	68.0%	32.0%
	2017	296,864	1.4%	53.0%	10.5%	2.9%	67.8%	32.2%
		Average	1.4%	51.7%	10.0%	4.8%	67.9%	32.1%
Six doses of monthly HWD or HWD combination preventive								
	2014	1,719,665	8.6%	24.2%	11.6%	0.2%	44.6%	55.4%
	2015	1,743,727	8.5%	24.3%	11.4%	0.3%	44.5%	55.5%
	2016	1,714,466	8.3%	24.3%	11.2%	0.3%	44.1%	55.9%
	2017	1,685,452	8.3%	24.6%	11.0%	0.4%	44.3%	55.7%
		Average	8.4%	24.4%	11.3%	0.3%	44.4%	55.6%

Monthly HWD products can be purchased early. Including early repurchases in the compliant category at 5–7 months increased the average purchase compliance for monthly HWD preventives to 32.8% (range 32.6–32.9%), which was still less than the average compliance for injectable moxidectin (Table 4).

### Revenue

Total revenue per dog from the two groups was an arithmetic mean, determined by adding all the invoices for each visit (i.e., visit 1 and visit 2) and dividing by the number of dogs. The average total invoice for the injectable moxidectin first visit was $161, and $135 for the second. In comparison, the average six-dose monthly preventive first visit revenue was $141, and the second six-dose visit revenue was $125. The average six-dose monthly combination preventive total invoice was $171 for the first visit and that of the second visit was $158 (Table 5). The higher average revenue from HWD combination products is not unexpected. Commensurate with their acquisition cost, most HWD combination products have a higher retail cost than standard HWD products. This is demonstrated in the next section.

**Table 5:** Comparison of total revenue and cost of HWD preventive between injectable moxidectin, monthly HWD preventives, and HWD combination preventives.

**Six-dose patients**	**Invoice year**	**Patient count**	**Average total spend**		**Average HWD spend**		**HWD spend as a proportion of total invoice**		**Average HWD spend/month or per dose**	
			**Visit 1**	**Visit 2**	**Visit 1**	**Visit 2**	**Visit 1**	**Visit 2**	**Visit 1**	**Visit 2**
Biannual injectable moxidectin										
	2014	90,831	$149	$121	$45	$46	30.2%	38.0%	$7.50	$7.67
	2015	100,407	$154	$133	$47	$48	30.5%	36.1%	$7.83	$8.00
	2016	129,916	$165	$140	$48	$50	29.1%	35.7%	$8.00	$8.33
	2017	157,225	$176	$148	$50	$51	28.4%	34.5%	$8.33	$8.50
Monthly HWD preventive										
	2014	194,236	$121	$107	$43	$44	35.5%	41.1%	$7.17	$7.33
	2015	215,669	$136	$120	$45	$46	33.1%	38.3%	$7.50	$7.67
	2016	239,278	$148	$131	$47	$47	31.8%	35.9%	$7.83	$7.83
	2017	259,219	$158	$141	$48	$48	30.4%	34.0%	$8.00	$8.00
Monthly HWD combination preventive										
	2014	207,021	$164	$150	$93	$94	56.7%	62.7%	$15.50	$15.67
	2015	193,255	$168	$154	$94	$95	56.0%	61.7%	$15.67	$15.83
	2016	166,170	$173	$160	$97	$98	56.1%	61.3%	$16.17	$16.33
	2017	147,040	$178	$167	$99	$100	55.6%	59.9%	$16.50	$16.67

### Cost of HWD Prevention

Injectable moxidectin was no more expensive to the owner than six doses of monthly HWD preventive. The average cost of injectable moxidectin per dog to the pet owner was $48 for visit 1 and $49 for visit 2, representing 29.7% of the total visit invoice (range 28.6–30.4%). The average cost of six doses of HWD prevention was $45 for visit 1 and $46 for visit 2, representing 31.0% of the total invoice (range 30–32%). In contrast, the average cost of six doses of combination HWD prevention was $95 for visit 1 and $97 for visit 2, representing 59% of the total invoice (Table 5).

Injectable moxidectin visits had a larger proportion of pet owners purchasing additional products and services with their veterinarian, with 85% of patients having additional transactions recorded during the first injectable moxidectin visit and 77% during the second visit. In contrast, only 55% of pet owners who purchased six doses of monthly HWD prevention also purchased additional products and services with their veterinarian during the first visit. Only 46% purchased additional products and services with their veterinarian during the second visit (Table 6).

**Table 6:** Proportion of pets with records of purchases additional to HWD prevention.

**Transaction type**	**Injectable moxidectin**		**Monthly HWD**		**Monthly HWD combination**	
	**Visit 1**	**Visit 2**	**Visit 1**	**Visit 2**	**Visit 1**	**Visit 2**
Additional products and services						
	84.7%	77.3%	55.6%	45.2%	54.7%	46.3%
Flea and tick	17.4%	14.2%	29.8%	29.1%	7.3%	6.9%
Pruritus product	4.5%	4.6%	3.4%	3.4%	3.8%	3.8%
NSAID	2.9%	2.5%	2.5%	2.3%	2.4%	2.2%
Anti-infective	4.3%	4.0%	3.3%	3.0%	3.5%	3.1%
Anti-emetic	0.6%	0.5%	0.6%	0.6%	0.7%	0.6%

### Patient Retention at a Veterinary Clinic

For patient retention, the injectable moxidectin cohort of 2014 had a higher proportion of patients with repeat injections within 12 months between 2014 and 2017, with 68% retention after 4 years. In comparison, the six-dose monthly HWD cohort's retention dropped to 55% by 2017. A similar trend was noted in dogs aged 6–9 months, with 62% injectable moxidectin retention after 4 years, compared with 41% for monthly preventives (data not shown). Therefore, injectable moxidectin demonstrated higher retention and compliance compared with monthly HWD (Table 7).

**Table 7:** Comparison of patient retention (6-month injectable moxidectin vs. monthly preventive, all dogs).

**2014 patient cohort product**	**Year**	**Patient count**	**Patients with 12 + total doses**	**% patients with 12 + total doses**	**Patient retention**
Biannual injectable moxidectin					
	2014	73,568			100%
	2015	65,682	41,243	63%	89%
	2016	56,386	38,780	69%	77%
	2017	50,361	35,249	70%	68%
Monthly HWD or HWD combination preventive					
	2014	637,897			100%
	2015	590,099	325,367	55%	93%
	2016	448,112	263,891	59%	70%
	2017	351,775	211,662	60%	55%

## Discussion

The goal of this study was to examine HWD preventive purchase compliance and economic value to the veterinary hospital. While pharmacy and food dispensing represent 26% of veterinary hospital income (14), consumers are increasingly purchasing products online when possible (15). Injectable moxidectin heartworm disease prevention ensures 100% compliance, achieves superior patient retention, and can only be sourced through the veterinary professional. Bringing the dog to the clinic for each injection offers additional opportunities for continued engagement with pet owners and reinforces the importance of HWD prevention. Furthermore, having prevention done by the veterinarian guarantees better health outcomes with regard to HWD control because it takes preventive compliance out of the hands of the owner.

Using AHS guidelines as a working definition for compliance, this study demonstrated that annual compliance with biannual injectable moxidectin was higher than purchase compliance with the exact dose-equivalent in monthly HWD preventives. The average proportion of dogs that were compliant for two injections of moxidectin 5–7 months apart was 51.7% (range 49.7–53.1%). In comparison, the number of dogs compliant with purchasing six doses of monthly HWD preventives 5–7 months apart was 24.4%. When we include second visit monthly purchases that were made early, the average compliance in this group rose to 32.8%, which was still less than the injectable form. Furthermore, there exists a proportion of pets that will receive their HWD prevention from their veterinarian only once each year. Thirty-two percent of moxidectin dogs (range 31.0–33.1%) were protected for only 6 months (i.e., got a single moxidectin injection in a 12-month period), compared with 55.6% of dogs with a single transaction for six doses of a monthly HWD or HWD combination product in a 12-month period.

Pet owner HWD preventive purchase compliance and administration has important health implications. For instance, Atkins et al. (11) demonstrated that in over 80% of HWD cases evaluated, insufficient HWD preventive doses were purchased, with purchase gaps of >45 days, which opened a potential window of exposure to and infection with HWD. In the current study, 32% of injectable moxidectin recipients and 56% of equivalently dosed monthly HWD preventive recipients were only covered for 6 months out of the year through their veterinarian. This presents potential compliance gaps contrary to AHS guidelines.

There appears to be a health benefit to HWD preventive purchase compliance, and these data may support the premise that dogs acquiring more doses of HWD preventive through their veterinarian are also examined more fully (16). The current study showed that 85% of injectable moxidectin recipients had records of purchasing additional products and services, other than flea and tick preventives, with their veterinarian during the first visit, and 77% did so during the second visit. Fifty-six percent of monthly preventive recipients purchased additional products and services for visit 1, and 45% for visit 2, or 55% of monthly preventive combination for visit 1, and 46% for visit 2. Because this study could not corroborate transactions with medical records, it was not possible to determine what services were purchased other than the broad categories listed in Table 6. However, the American Animal Hospital Association (AAHA) Financial and Productivity Pulsepoints (14) suggests that general health expenditure in veterinary care is broken down as follows: dispensing, 18.8%; food, 4.6%; OTC, 5.5%; laboratory, 16.7%; outpatient, 18.0%; vaccination, 9.4%; anesthesia, 3.2%; surgery, 7.9%; imaging, 4.1%; dental, 3.8%; inpatient, 2.8%; and other, 5.2%. This study also supports the premise that injectable moxidectin administration provides additional opportunities for health assessments compared with monthly HWD preventives (16).

The current study also examined the cost of HWD preventives to the pet owner. On average, injectable moxidectin was no more expensive than six doses of monthly HWD prevention. The average cost of biannual injectable moxidectin per dog was $48 for visit 1 and $49 for visit 2. The average cost of six doses of monthly HWD prevention was $45 for visit 1 and $46 for visit 2. No peer-reviewed studies have been published on the cost of heartworm treatment in dogs, but the AHS estimates the average cost of treating a dog already diagnosed with heartworm at $1,200–$1,800 (available at https://d3ft8sckhnqim2.cloudfront.net/images/infographics/0010-weigh-the-costs.jpg).

Finally, clients purchasing injectable moxidectin showed a higher proportion of patients loyal to this preventive modality, with 68% retention over the 4 years observed. In comparison, monthly HWD loyalty (retention) dropped to 55% by 2017. Retention will very likely be easier to achieve with the annual preventive option of ProHeart® 12 because clients will not be required to return every 6 months to be considered compliant.

This research suggests that clients who purchased 6-month injectable moxidectin vs. monthly preventives demonstrated significantly better compliance (falling closer in line with AHS recommendations) and superior retention over the 4-year study period. It is likely that these factors will rise with the now available ProHeart® 12, which, at similar cost, guarantees 100% consumer compliance for the full AHS-recommended 12 months after administration and does not require owners to purchase a second dose every 6 months.

### Limitations

In this study, we utilized transaction records for the time frame 2014–2017 of products and services purchased by clients. Examination of clinic transaction records is an imperfect substitute for estimating the number of medication doses administered to a pet (17). However, transactional data are an objective measure of pet owner adherence to veterinary recommendations (at least through their purchase of recommended product) unlike owner self-reported adherence which may be biased or unreliable (17). It is known that people are highly non-compliant with their own prescribed medications as well as administering them to dependent family members (18–21). Furthermore, monthly HWD products can be purchased in smaller quantities multiple times in the year and could be shared among dogs from multiple dog households. Thus, true compliance in administering monthly HWD preventives to pet dogs was not evaluated in this study, only purchase compliance. While the focus of the study was on veterinarian-sourced HWD preventive compliance, this study does not account for potential prescriptions of monthly HWD preventives that owners may have filled through online pharmacies. These missing data could potentially alter the results of this compliance comparison.

## Conclusions

We conclude that 6-month injectable moxidectin was superior in HWD preventive compliance to monthly products. Injectable moxidectin was associated with better revenue-generating potential, with up to 85% of clients receiving injectable moxidectin purchasing additional products and services from the clinic. Injectable moxidectin, now available in 12-month extended release form, has the potential to improve the veterinary profession's battle against lack of compliance for this often fatal, yet preventable, canine infectious disease.

## Data Availability Statement

The raw data supporting the conclusions of this article will be made available by the authors, without undue reservation.

## Author Contributions

KM was responsible for the study design, analysis, interpretation, and manuscript development. LS, MM, and CB were responsible for the analysis, production of descriptive summaries, and data visualization. CW and KB were responsible for the interpretation and manuscript development. All authors read and approved the final manuscript.

## Conflict of Interest

KM, DA, CW, and KB are employed by Zoetis Inc. The authors declare that this study received funding from Zoetis. The funder had the following involvement with the study: study design and data analysis. The remaining authors declare that the research was conducted in the absence of any commercial or financial relationships that could be construed as a potential conflict of interest.

## Abbreviations

AHS: American Heartworm Society
HWD: heartworm
US: United States.
